# Phosphodiesterase *SMPDL3B* Gene Expression as Independent Outcome Prediction Marker in Localized Prostate Cancer

**DOI:** 10.3390/ijms21124373

**Published:** 2020-06-19

**Authors:** Frank Waldbillig, Katja Nitschke, Abdallah Abdelhadi, Jost von Hardenberg, Philipp Nuhn, Malin Nientiedt, Cleo-Aron Weis, Maurice Stephan Michel, Philipp Erben, Thomas Stefan Worst

**Affiliations:** 1 Department of Urology and Urosurgery, University Medical Centre Mannheim, University of Heidelberg, 68167 Mannheim, Germany; katja.nitschke@medma.uni-heidelberg.de (K.N.); aabdelhadi92@gmail.com (A.A.); jost.vonhardenberg@medma.uni-heidelberg.de (J.v.H.); Philipp.Nuhn@medma.uni-heidelberg.de (P.N.); malin.nientiedt@umm.de (M.N.); maurice-stephan.michel@umm.de (M.S.M.); philipp.erben@medma.uni-heidelberg.de (P.E.); Thomas.Worst@umm.de (T.S.W.); 2 Institute of Pathology, University Medical Centre Mannheim, University of Heidelberg, 68167 Mannheim, Germany; Cleo-Aron.Weis@umm.de

**Keywords:** cancer cell migration, prognosis, biomarker, extracellular vesicles, lipid metabolism

## Abstract

Current outcome prediction markers for localized prostate cancer (PCa) are insufficient. The impact of the lipid-modifying Sphingomyelin Phosphodiesterase Acid Like 3B (SMPDL3B) in PCa is unknown. Two cohorts of patients with PCa who underwent radical prostatectomy (*n* = 40, *n* = 56) and benign prostate hyperplasia (BPH) controls (*n* = 8, *n* = 11) were profiled for *SMPDL3B* expression with qRT-PCR. Publicly available PCa cohorts (Memorial Sloane Kettering Cancer Centre (MSKCC; *n* = 131, *n* = 29 controls) and The Cancer Genome Atlas (TCGA; *n* = 497, *n* = 53 controls)) served for validation. SMPDL3B’s impact on proliferation and migration was analyzed in PC3 cells by siRNA knockdown. In both cohorts, a Gleason score and T stage independent significant overexpression of *SMPDL3B* was seen in PCa compared to BPH (*p* < 0.001 each). A lower expression of *SMPDL3B* was associated with a shorter overall survival (OS) (*p* = 0.005) in long term follow-up. A *SMPDL3B* overexpression in PCa tissue was confirmed in the validation cohorts (*p* < 0.001 each). In the TCGA patients with low SMPDL3B expression, biochemical recurrence-free survival (*p* = 0.011) and progression-free interval (*p* < 0.001) were shorter. Knockdown of *SMPDL3B* impaired PC3 cell migration but not proliferation (*p* = 0.0081). In summary, *SMPLD3B* is highly overexpressed in PCa tissue, is inversely associated with localized PCa prognosis, and impairs PCa cell migration.

## 1. Introduction

Prostate cancer (PCa) is the most common solid tumor entity in men in developed countries [1,2]. Most tumors are detected in early stages and then, tend to show low aggressiveness with a slow tumor growth. Some of these patients probably do not need radical therapy. Aggressive PCa tumors on the other hand quickly metastasize to regional lymph nodes and the skeleton. This results in a fatal disease state that requires a long and demanding therapy. Therefore, it is of paramount priority to identify those patients in need of definite local treatment and those which can be spared from overtreatment.

Reliable PCa markers are needed both for therapy decision-making and risk prediction of tumor recurrence after curatively intended therapy, as the currently available tools still have significant deficiencies. In a recent study, we identified a number of potential protein biomarkers for high-risk PCa by proteomic profiling of PCa cells and their extracellular vesicles (EVs) combined with a screening of publicly available databases [3]. EVs have shown to be of great relevance for multiple tumor-associated processes such as local tumor invasion, induction of neoangiogenesis, and premetastatic niche formation in different tumor entities including PCa [4,5]. Screening of potential markers revealed that the SMPDL3B (acid sphingomyelinase-like phosphodiesterase 3B) protein is associated with PCa-derived EVs. Gonzales et al. have already detected the SMPDL3B protein among hundreds of others in human urinary EVs [6]. Principe et al. could detect the protein in analyses of EV’s isolated from expressed prostate secretions in human urine [7]. Furthermore, our database analyses indicated an overexpression of the coding SMPDL3B gene in PCa tissue [3].

The SMPDL3B protein is an important enzyme for the lipid-modulation of the cell membrane and thus influences, e.g., the membrane fluidity [8]. So far, nothing has been published about the role of SMPDL3B in PCa. Recently, it was reported that SMPDL3B has impact on podocyte function in renal glomeruli. Thus, the SMPDL3B expression rate in vitro and in vivo correlates with podocyte damage in diabetic kidney disease (DKD) [9]. Apparently, insulin receptor signaling in podocytes in DKD is disturbed via the SMPDL3B-mediated reduction of ceramide-1-phosphate [10]. Watanabe et al. identified SMPDL3B as potential marker for therapeutic response in rituximab-based immuno-suppressive therapy in pediatric patients with intractable kidney disease with proteinuria [11]. This approach is mainly based on the findings of Heinz et al., who first described SMPDL3B as a negative regulator of innate immunity via reduction of Toll-like receptor function on macrophages [8].

Based on our previous findings we aimed to elucidate the role of SMPDL3B on the transcription level. Therefore, SMPDL3B expression and its influence on clinical outcome was analyzed by real-time quantitative polymerase chain reaction (qRT-PCR) in two cohorts of patients with benign prostate hyperplasia (BPH), localized and locally advanced PCa. Furthermore, these results were validated in current, publicly available PCa expression data sets, and in vitro analyses were performed in a PCa cell line.

## 2. Results

### 2.1. qRT-PCR Analyses in Patients

**Figure 1:**
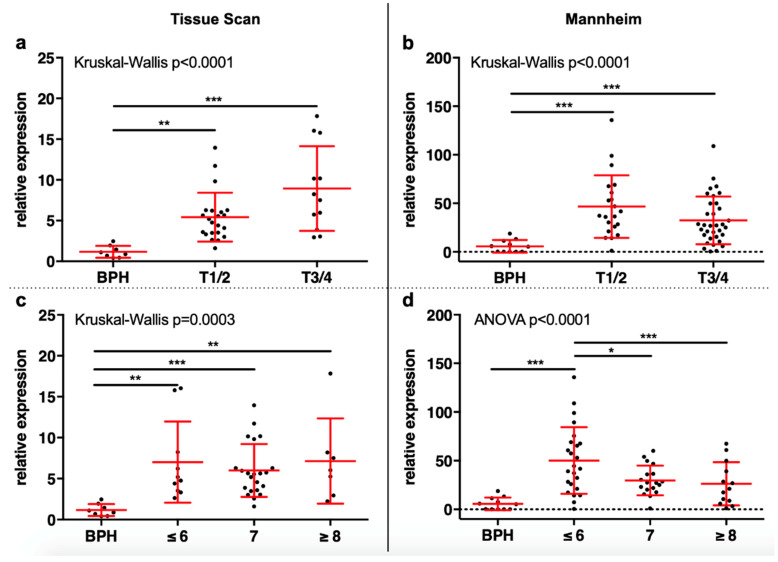
Expression of Sphingomyelin Phosphodiesterase Acid Like 3B (SMPDL3B) was analyzed by qRT-PCR. (**a**) Both locally confined and locally advanced tumors showed an overexpression of SMPDL3B compared to benign prostate hyperplasia (BPH). (**b**) In the Mannheim cohort, T1/2 and T3/4 tumors had an SMPDL3B overexpression. (**c**) All three Gleason groups had an SMPDL3B overexpression in the tissue scan cohort. (**d**) In the Mannheim cohort, Gleason 6 tumors had an overexpression of SMPDL3B both compared to BPH and to Gleason 7 and ≥ 8 tumors. (* *p* > 0.05; ** *p* > 0.01; *** *p* > 0.001)

In both cohorts analyzed with qRT-PCR a significant overexpression of SMPDL3B could be seen in tumor samples compared to BPH samples (Tissue scan: 6.46× and Mannheim: 37.81×, Mann–Whitney for both *p* < 0.001). This was also seen after stratification of tumor samples for T stage (both *p* < 0.001, Figure 1a,b) and Gleason score (both *p* < 0.001, Figure 1c,d). In the tissue scan cohort, multiple comparison showed significant overexpression of SMPDL3B in both locally confined (T1/2: 5.42×, *p* = 0.001) and locally advanced (T3/4: 8.94×, *p* < 0.001) PCa and in different Gleason groups (≤ 6: 7.01×, *p* = 0.002; 7: 6.00×, *p* < 0.001; ≥ 8: 7.14×, *p* = 0.004) compared to BPH. No significant differences were seen between tumor groups. Similar results were seen in the Mannheim cohort: T1/2 vs. BPH: 46.6× (*p* < 0.001), T3/4 vs. BPH: 32.37 (*p* < 0.001), Gleason ≤ 6 vs. BPH: 50.19 (*p* < 0.001). Controversially, no significant overexpression in Gleason 7 or Gleason ≥ 8 compared to BPH was seen, while the expression was also significantly higher in Gleason ≤ 6 tumors compared to Gleason 7 (*p* = 0.045) and Gleason ≥ 8 (*p* = 0.030) tumors. In the Mannheim cohort, SMPDL3B expression did not correlate with the serum PSA level (Spearman *r* =−0.103, *p* = 0.453). For the tissue scan cohort, no serum PSA data were available.

**Figure 2:**
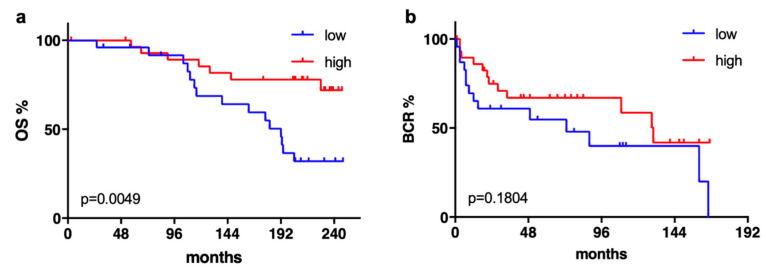
(**a**) In the Mannheim cohort, a low expression of SMPDL3B correlated with a significantly shorter overall survival (OS) but (**b**) not with a shorter biochemical recurrence (BCR)-free survival of patients with localized prostate cancer (PCa) who underwent RP.

Interestingly, after a 50:50 division of the Mannheim cohort into two groups by SMPDL3B expression, a lower expression of SMPDL3B in tumor samples correlated with a shorter OS (Figure 2a, *p* = 0.005) in long-term follow-up (average follow-up time: 168 months). Using the same cutoff, no significant difference was seen for BCR (Figure 2b).

### 2.2. In Silico Validation

**Figure 3:**
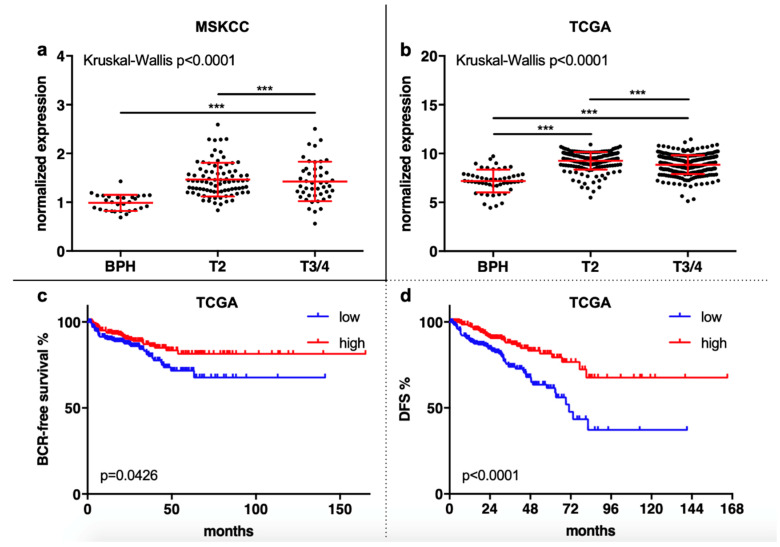
(**a**) and (**b**) Both in the Memorial Sloane Kettering Cancer Centre (MSKCC) and in The Cancer Genome Atlas (TCGA) cohort, the expression of SMPDL3B was higher in T2 and in T3/4 tumors compared to BPH. In the TCGA cohort, the expression was also significantly higher in T2 compared to T3/4 tumors. (**c**) and (**d**) A low expression of SMPDL3B was associated with a significantly shorter BCR-free survival and progression-free interval (PFI) in the PCa patients from the TCGA cohort. (*** *p* > 0.001)

In silico analyses confirmed the overexpression of SMPDL3B in PCa tissue in the MSKCC dataset (*p* < 0.001) and in the TCGA dataset (*p* < 0.001). In the MSKCC dataset, this was both seen for T2 and T3/4 tumors (both *p* < 0.001), with no differences in expression between T2 and T3/4 tumors (Figure 3a). In the TCGA cohort, besides a higher expression in T2 and T3/4 compared to BPH (both *p* < 0.001), interestingly, also a significantly higher expression in T2 compared to T3/4 tumors was seen (*p* < 0.001, Figure 3b).

In the TCGA cohort, the SMPDL3B expression correlated positively with the expression of KLK3, the gene which codes for PSA, (*r* = 0.287, *p* < 0.001) and correlated negatively with the expression of *AR* (*r* = −0.148, *p* < 0.001) and the Gleason sum score (*r* =−0.336, *p* < 0.001). Again, with a 50:50 SMPDL3B expression cut-off in the MSKCC cohort, no difference was seen for BCR-free survival and OS. Yet, the number of events in this cohort is quite small (only 26 cases with BCR and only 7 deceased patients). In the TCGA cohort, also no significant difference was seen for OS (only *n* = 10 patients deceased in total), but a low expression in tumor samples correlated with a significantly shorter BCR-free survival (*p* = 0.043) and a shorter progression-free interval (*p* < 0.001) (Figure 3c,d).

### 2.3. Functional Experiments

**Figure 4:**
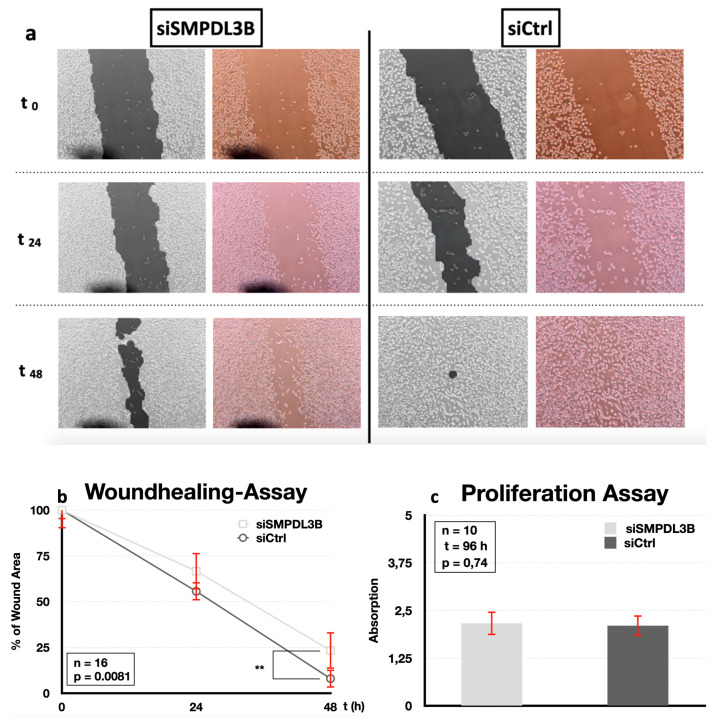
(**a**) Wound-healing migration assay in PC3 cells after siRNA knockdown of SMPDL3B (*n* = 16). Images were taken at 0, 24, and 48 h after scratch. Calculation of the wound area was performed using TScratch software^®^ (black and white images), with a percentage of the wound area being given for each image. (**b**) Quantitative results of the migration assay which showed a significant reduction of the migration of siSMPDL3B-transfected cells compared to siCtrl after 48 h (*p* = 0.0081). (** *p* > 0.01) (**c**) PC3 cell proliferation after siRNA knockdown was not significantly altered after knockdown of SMPDL3B (*n* = 10, *p* = 0.74).

The siRNA knockdown of SMPDL3B significantly impaired migration of PC3 cells compared to nontargeting siCtrl (*n* = 16). In brief, 24 h after application of the defect, the wound area of the cells treated with siSMPDL3B was 0.666 ± 0.107 compared to siCtrl at 0.556 ± 0.236 (*p* = 0.98). After 48 h of growth, the wound area of the cells treated with siSMPDL3B was 0.264 ± 0.116 compared to siCtrl at 0.079 ± 0.054, (*p*= 0.0081, Figure 4a,b).

However, the siRNA knockdown of SMPDL3B had no significant effect on PC3 cell proliferation after 96 h in *n* = 10 replicates compared to siCtrl (2.165 ± 0.312 vs. 2.103 ± 0.273, respectively) (Figure 4c).

## 3. Discussion

PCa development is highly heterogenetic and a multifactorial process with intracellular dysregulations on several molecular levels [12]. Although these mechanisms are gaining a better understanding, it is probably the PCa diversity that has prevented potential tumor progression markers from being reliable. A little-noticed factor that plays an important role in cancer signaling is the lipid-based cell membrane [13]. In addition to its central role as boundary and stabilizer of cells, cell membranes can present different cancer-specific surface structures such as proteins and carbohydrates. In this study, we focused on the GPI membrane-anchored phosphodiesterase SMPDL3B. This, to date, little described protein is an enzymatic component of the lipid rafts of cell membranes and appears to be involved in a wide variety of molecular processes including the sphingomyelin metabolism [8]. Besides its relevance for membrane fluidity and lipid composition, SMPDL3B has been identified as a negative regulator of the innate immune signaling [14]. In addition, SMPDL3B overexpression reduces cell apoptosis and strengthens the actin skeleton.

Yet, little is known about the role of SMPDL3B in malignant diseases. In context of PCa, it has only been described in one study so far. By coincidence, Zhu et al. found SMPDL3B overexpression in docetaxel-resistant PC3 cells in a microarray analysis [15].

To our knowledge, this is the first study in which a significant overexpression of SMPDL3B in PCa tissue compared to nonmalignant prostate tissue could be demonstrated in two different patient cohorts. Since this overexpression was independent of the serum PSA level, SMDPL3B could potentially serve as an additional PCa screening marker. Curiously, we were able to show that a lower SMPDL3B expression in PCa was associated with a shorter OS but not with a shorter BCR-free survival. In silico validation in two freely available PCa cohorts (MSKCC, TCGA) confirmed a general SMPDL3B overexpression in patients with PCa. While the MSKCC cohort also showed no correlation of SMPDL3B expression with BCR-free survival, the TCGA cohort indicated that low SMPDL3B expression was associated with shorter BCR-free survival. This is in line with our findings of a stage-dependent, higher SMPDL3B expression in T2 tumors compared to T3/4 tumors. Interestingly, recent studies show that an excess of SMPDL3B leads to a decrease in ceramide-1-phosphate (C1P), a bioactive sphingolipid metabolite regulating key physiologic cell functions. In turn, C1P has been shown to interfere in cancer cell growth, migration, and survival [16]. One gene expression study described a downregulation of SMPDL3B during the mesenchymal to epithelial transition [17]. This might partly explain the inverse association of SMPDL3B expression with PCa patient prognosis.

Both the mechanisms regulating SMPDL3B expression and function and its main substrates remain unclear. However, SMPDL3B can cleave choline-containing substrates including CDP-choline and release phosphate from ATP and ADP. Furthermore, amino acid residues close to the active center of SMPDL3B have been identified. These can recognize endogenous molecules that modulate macrophage function [14]. Studies could show that decreased SMPDL3B levels lead to membrane disorder and a TLR-moderated hyperinflammatory cell phenotype [14]. One could speculate that high concentrations of SMPDL3B lead to a higher membrane stability and thus less disruption of cytoskeleton-lipid raft assemblies promoting carcinogenesis [18]. At the same time, its higher occurrence would reduce the inflammatory environmental reaction and thus reduce the cancer-specific immune defense.

The siRNA knockdown of SMPDL3B lead to a decrease in tumor cell migration. No effects on the proliferation rate were found. This may be explained by the important role of SMPDL3B for membrane stability. However, additional functional studies are required to confirm these results.

To the best of our knowledge, the present study for the first time showed that SMPDL3B is overexpressed in PCa independent of PSA, with higher expression levels in early stages. Its correlation with other clinical parameters promotes its potential applicability in screening and outcome prediction of PCa.

Excitingly, rituximab (RTX), a well-established monoclonal antibody against cell membrane-based CD20, which is used, e.g., in the treatment of malignant hematological diseases, also binds to SMPDL3B [19]. Different case reports have already shown a potential clinical use of RTX in the treatment of metastatic PCA [20,21,22]]. RTX binds to SMPDL3B in a CD20-independent mechanism [23], which leads to a stabilization of the SMPDL3B concentration in the raft domains and thus, to the stabilization of the cell membrane. Based on this, it could be shown that RTX-treated cells are less radiation sensitive [24]. Considering a modification of the antibody-structure presupposed, one could possibly revert this effect for a combination with PCa radiotherapy. Presumably, an antibody-mediated decrease in membrane-anchored SMPDL3B would lower the membrane stability and could render PCa cells more susceptible to external radiation.

SMPDL3B is a promising biomarker candidate, with potential value for screening and outcome prediction in PCa and at the same time could be an easily accessible target structure in PCa therapy.

## 4. Materials and Methods

### 4.1. Patient Characteristics

The expression of SMPDL3B was analyzed by qRT-PCR in a complementary deoxyribonucleic acid (cDNA) array (OriGene, Rockville, MD, USA; *n* = 40 PCa patients and *n* = 8 benign control samples, patient characteristics Table S1) and a cohort of 55 patients who underwent radical prostatectomy (RP) in the Department of Urology and Urosurgery of the Mannheim Medical Center between 1998 and 2001 (patient characteristics in Table S2). Tumor-free prostate tissue from patients who underwent cystoprostatectomy or transurethral resection of the prostate served as controls (*n* = 11). All experiments conducted in this retrospective analysis were in accordance with the institutional ethics review board (ethics approval date 05.12.2013, ethics number: 2013-845R-MA). The study includes retrospective analysis of patient data and qRT-PCR analysis of patient tissue samples obtained during surgery for routine pathologic examination. All analyses and experiments were approved by the local ethic committee (Medical Ethics Committee II of the Medical Faculty Mannheim; ethic approval: 2013-845R-MA).

The Memorial Sloane Kettering Cancer Centre (MSKCC, *n* = 131, *n* = 29 controls, Table S3) [25] and The Cancer Genome Atlas (TCGA, *n* = 497, *n* = 53 controls, Table S4) [26] were obtained from cBioPortal [27], and the University of California, Santa Cruz Xena Browser [28] served for in silico validation. SMPDL3B RNA expression was stratified by tumor characteristics, i.e., serum prostate specific antigen (PSA) levels and was correlated with biochemical recurrence (BCR)-free survival, progression-free survival (PFS), and overall survival (OS).

### 4.2. RNA Extraction and qRT-PCR Analyses

Hematoxylin- and eosin-stained sections of tumor-bearing or tumor-free formalin-fixed paraffin-embedded (FFPE) prostate tissue specimen of the Mannheim cohort were reviewed by a trained uropathologist. Areas with at least 70% of tumor or tumor-free areas from control patients were marked and dissected from subsequent unstained 10 μm tissue sections. Ribonucleic acid (RNA) was extracted using the bead-based XTRAKT FFPE Kit (Stratifyer, Cologne, Germany), as recommended by the manufacturer and recently published [29].

Multiplexed specific cDNA synthesis with equimolar pooling of transcript-specific reverse PCR primers was used. Superscript III (Life Technologies, Darmstadt, Germany) was used as reverse transcriptase at 55 °C for 120 min, followed by an incubation at 70 °C for 15 min. cDNA was immediately used for qPCR or stored at −20 °C.

In the cDNA array and the Mannheim cohort, the expression of SMPDL3B was determined in relation to the housekeeping gene calmodulin 2 (CALM2) on a StepOnePlus qRT-PCR Cycler (Applied Biosystems, Waltham, MA, USA) (all sequences in Table S5). Relative expression was calculated with the 2^(−ΔΔ*C*T)^ method [30]. The methodology was recently published elsewhere [29]. The sequences of primers and probes are given in Supplementary Table S1.

### 4.3. Small Interfering RNA (siRNA) Knockdown

The impact of SMPDL3B siRNA knockdown on proliferation and migration was analyzed in humane metastatic PC3 prostate cancer cells. These were obtained from ATCC (Wesel, Germany) and grown under standard conditions in DMEM medium (Life Technologies, Carlsbad, CA, USA) supplemented with 10% FCS (Sigma Aldrich, St. Louis, LA, USA). siGENOME pooled and individual siRNAs against SMPDL3B were transfected using DharmaFECT I transfection reagent (Dharmacon, Lafayette, CO, USA) (siSMPDL3B). Dharmacon nontargeting siRNAs were used as negative control (siCtrl). Briefly, cells were detached, harvested, spun down, and diluted to the desired concentration. Meanwhile siRNAs were diluted to a target concentration of 30 nMol in pure RPMI (Life Technologies) and incubated for 10 min at room temperature. DharmaFECT I was diluted 1:1000 in RPMI medium. After 10 min, diluted siRNA and transfection reagent were mixed (1:1) and again incubated at room temperature for 30 min. Hereafter, cell suspension was added to the transfection mix (3:1) and incubated at 37 °C.

qRT-PCR was conducted to validate knockdown SMPDL3B. RNA extraction was performed using the RNeasy Mini Kit (Qiagen, Hilden, Germany) as recommended by the manufacturer. cDNA synthesis was performed as described previously [31]: in brief, 40 μL of diluted RNAs were mixed with 4 μL of 5 mg/mL pdN6 random primers, 4 μL of 10 mM dNTP Mix, 16 μL of 5× M-MLV buffer, 8 μL of 0.1 M RNase inhibitor, 4 μL of 0.1 M DTT, and 4 μL of M-MLV reverse transcriptase (all from Roche Diagnostics, Basel, Switzerland). After an incubation for 2 h at 37 °C and a deactivation step of 5 min at 65 °C, cDNA was directly used for qPCR or stored at −20 °C. qRT-PCR analyses were performed using the same primers, reagents, and PCR protocol as described for tissue sample analyses.

### 4.4. Proliferation Assay

PC3 cells were seeded and transfected in 96-well plates (4500 cells in 100 μL/well). After 24 h, the supernatant was replaced by 100 μL of fresh growth medium. After further 96 h of incubation, 10 μL of MTT-reagent (stock concentration 1.585 mg/mL, product number G3582, Promega, Mannheim, Germany) was added to each well and incubated for 3 h at 37 °C. Absorption measurement at 570 nm was done with an Infinite M1000 PRO plate reader (Tecan, Männedorf, Switzerland).

### 4.5. Migration Assay

Using the same transfection protocol, a wound-healing migration assay of PC3 cells was performed. The cells were seeded in 24-well plates (250,000 cells in 1 mL of DMEM with 10% FCS per well). The medium was changed 24 h after transfection. Again 24 h later, a defined scratch was introduced in the center of the well with a sterile 200 μL pipette tip, and the medium was changed again. The scratch was photographed at 10× magnification. Subsequent images were acquired after 24 and 48 h. The cell-free space in the scratch area was calculated with the open source software TScratch (ETH Zürich, Switzerland) [32]. The free area at 24 and 48 h after scratch was normalized to the initial scratch size.

### 4.6. Statistics

Statistical calculations were performed using Prism 7 (GraphPad, La Jolla, CA, USA). Different subgroups were tested for normal distribution using the D’Agostino-Pearson test for normal distribution. In case of a normal distribution, subsequent group comparisons were performed by two-sided *t*-test or parametric ANOVA with subsequent Turkey’s post hoc multiple comparison tests. In case of non-normal distribution Mann–Whitney test or Kruskal–Wallis test with Dunn’s post hoc multiple comparison tests were performed. Outcome correlations in Kaplan–Meier analyses were done using the log-rank test. Correlations with the PSA serum level were performed using Spearman correlation. *p*-values ≤ 0.05 were deemed significant.

## Supplementary Materials

Supplementary materials can be found at https://www.mdpi.com/1422-0067/21/12/4373/s1. Table S1: Characteristics of the purchased cohort; Table S2: Characteristics of the Mannheim cohort; Table S3: Characteristics of the MSKCC cohort; Table S4: Characteristics of the TCGA cohort; Table S5: Primers & Probes.
