# Supplementary material for: Phosphodiesterase SMPDL3B Gene Expression as Independent Outcome Prediction Marker in Localized Prostate Cancer
Source: Int J Mol Sci. 2020 Jun 19;21(12):4373. doi: 10.3390/ijms21124373 (PMC7352472; doi:10.3390/ijms21124373)
Supplement: Supplementary file 1 [file ijms-21-04373-s001.pdf]

International Journal of
Molecular Sciences

MDPI

Supplementary Materials

Table S1. Characteristics of the cohort of purchased RNA material.

**Parameter**	***n***
Patients with PCa	40 (mean age 62.8 years, range 61–87)
T Stage	
T1	-
T2	22
T3	12
T4	-
n/a	6
N Stage	
N0	20
N1	2
Nx	18
Gleason Score	
5	2
6	8
7a	14
7b	8
8	3
9	4
n.a.	1
Control patients	8 (mean age 64.0 years, range 48–76)

Table S2. Characteristics of the patients who underwent radical prostatectomy (RP) at our own institution.

**Parameter**	***n***
Patients with PCa	55 (mean age 62.7 years, range 41–78)
T Stage	
T1	-
T2	21
T3	31
T4	3
N Stage	
N0	43
N1	5
N2	7
Gleason Score	
5	10
6	14
7a	14
7b	4
8	4
9	3
10	2
n.a.	4
Average serum PSA level	13.3 ng/mL (2.8–73.0 ng/mL)
Control patients	11 (mean age 66.8, range 49–77)

Table S3. Patient characteristics of the Memorial Sloane Kettering Cancer Centre (MSKCC) cohort.

Parameter	*n*
Patients with PCa	131 (mean age 58.0 years, range 47–83)
T Stage	
T1	-
T2	85
T3	40
T4	6
N Stage	
N0	99
N1	5
N2	27
Gleason Score	
6	41
7a	53
7b	21
8	8
9	7
10	0
n.a.	1
Average serum PSA level	8.6 ng/mL (1.2–46.3 ng/mL)
Adjacent tumor-free tissue samples	29 (mean age 56.7, range 49–66)

Table S4. Characteristics of the patients analyzed from The Cancer Genome Atlas (TCGA) cohort.

Parameter	*n*
Patients with PCa	497 (mean age 61.0 years, range 41–78)
T Stage	
T1	-
T2	187
T3	293
T4	10
n.a.	7
N Stage	
N0	345
N1	79
N2	73
Gleason Score	
6	45
7	247
8	64
9	137
10	4
Adjacent tumor-free tissue samples	53 (mean age 60.3 range 43–72)

Table S5. Primers and Probes.

**Primers**			
**Name**	**Primer Pair**	**Sequence (5′–3′)**	**Amplicon Length**
CALM2	Forward	GAGCGAGCTGAGTGGTTGTG	72 nt
	Reverse	AGTCAGTTGGTCAGCCATGCT	
SMPDL3B	Forward	CGCTGACAGCAGACATGG	69 nt
	Reverse	GTGGCCGACAATGTACACC	
**Probes**			
**Name**	**Probe Sequence/Name**		
CALM2	TCGCGTCTCGGAAACCGGTAGC		
SMPDL3B	UPL #11 (Roche, Diagnostics Mannheim)		
**siRNA**			
SMPDL3B	No 1: AGAGAGGTCTTTCCAGATA		
	No 2: GTAACAACATCTACAATCA		
	No 3: TGGAAACCCTGGCTTAGTA		
	No 4: GCTGGGAGCTCGAGTACCA		
